# Medullary and papillary carcinoma of the thyroid gland occurring as a collision tumor with lymph node metastasis: A case report

**DOI:** 10.1186/1752-1947-5-590

**Published:** 2011-12-20

**Authors:** Mehr Sadat Alavi, Negar Azarpira

**Affiliations:** 1 Department of Nuclear Medicine, Shiraz University of Medical Sciences, Shiraz, Iran; 2 Department of Pathology, Organ Transplant Research Center, Shiraz University of Medical Sciences, Shiraz, Iran

## Abstract

**Introduction:**

Papillary thyroid carcinoma and medullary thyroid carcinoma are two different thyroid neoplasia. The simultaneous occurrence of medullary thyroid carcinoma and papillary thyroid carcinoma as a collison tumor with metastases from both lesions in the regional lymph nodes is a rare phenomenon.

**Case presentation:**

A 32-year-old Iranian man presented with a fixed anterior neck mass. Ultrasonography revealed two separate thyroid nodules as well as a suspicious neck mass that appeared to be a metastatic lesion. The results of thyroid function tests were normal, but the preoperative calcitonin serum value was elevated. Our patient underwent a total thyroidectomy with neck exploration. Two separate and ill-defined solid lesions grossly in the right lobe were noticed. Histological and immunohistochemical studies of these lesions suggested the presence of medullary thyroid carcinoma and papillary thyroid carcinoma. The lymph nodes isolated from a neck dissection specimen showed metastases from both lesions.

**Conclusions:**

The concomitant occurrence of papillary thyroid carcinoma and medullary thyroid carcinoma and the exact diagnosis of this uncommon event are important. The treatment strategy should be reconsidered in such cases, and genetic screening to exclude multiple endocrine neoplasia 2 syndromes should be performed. For papillary thyroid carcinoma, radioiodine therapy and thyroid-stimulating hormone suppressive therapy are performed. However, the treatment of medullary thyroid carcinoma is mostly radical surgery with no effective adjuvant therapy.

## Introduction

Papillary thyroid carcinoma (PTC) and medullary thyroid carcinoma (MTC) are two different thyroid neoplasia. The former originates from thyroglobulin-producing follicular cells, whereas the latter arises from calcitonin-producing cells. MTC is a rare tumor that arises from neural crest-derived parafollicular C cells. Tumors showing both features are rare and represent less than 1% of all thyroid malignancies [1] and have different patterns of clinical presentation and biological behavior. A review of the literature revealed similar lesions [1-11]. Mutations in the RET gene, rearrangements of the tyrosine kinase receptors RET (ret/PTC) and NTRK1, and point mutation of the BRAF gene have been documented in PTC tumors [12-15]. However, the exact pathogenesis of these cases is unknown, but an underlying genetic background has been hypothesized. In this report, a case of concurrent MTC and PTC has the features of a collision tumor.

## Case presentation

A 32-year-old Iranian man was admitted for an anterior neck mass. He had no history of radiation to the head and neck and no known family history of endocrine disease. During a physical examination, his nodule was well demarcated, fixed, and measured about 2.5 cm in its largest diameter. An ultrasound examination of his neck showed a 3 cm solid hypoechoic nodule of the right thyroid lobe and an additional nodule in the lower pole of the same lobe. A suspicious neck mass that appeared to be metastatic lymph nodes was noticed. The left thyroid lobe and the isthmus appeared to be normal. Serum levels of free triiodothyronine, free thyroxine, and thyrotrophin were within normal ranges, and anti-thyroperoxidase/anti-thyroglobulin autoantibodies and anti-thyroglobulin antibody (anti-Tg Ab) were negative. A preoperative calcitonin serum value was elevated (40 ng/L) (chemiluminescence immunometric assay kit with reference intervals in adults: less than 11.5 ng/L for men and less than 4.6 ng/L for women). Our patient was screened for multiple endocrine neoplasia with negative results. He had normal serum levels of calcium, phosphorus, and parathyroid hormone. A chest X-ray and abdominal ultrasound were unremarkable. Fine needle aspiration of one nodule revealed papillary clusters that had atypical nuclei and intranuclear inclusions and that appeared to be a papillary carcinoma. Our patient underwent a total thyroidectomy with neck exploration. An enlarged thyroid gland with a prominent right lobe as well as a mottled lymph node in the right side were detected during the operation. Surgical specimens were fixed in 10% buffered formalin, embedded in paraffin, and stained with hematoxylin and eosin. For immunohistochemical studies, sections were incubated with the following primary monoclonal antibodies: cytokeratin AE1/AE3, chromogranin A, synaptophysin, thyroglobulin, and calcitonin (Dako Corporation, Glostrup, Denmark). An EnVision Dual Link system-HRP (ready to use; Dako Corporation) was used as a secondary antibody. Incubation with DAB (3,3'-diaminobenzidine tetrahydrochloride) was performed as a substrate chromogen solution to produce a brown color. All steps were carried out at room temperature. Appropriate positive and negative control sections were processed in parallel. An ill-defined, solid, tan-colored lesion measuring 3 cm in its greatest diameter was observed grossly in the right lobe. In the same lobe, an irregular, whitish lesion measuring 1 cm in its greatest diameter was also present and was completely separated from the former lesion. The remaining thyroid tissue was unremarkable. A neck dissection yielded eight regional lymph nodes.

**Figure 1:**
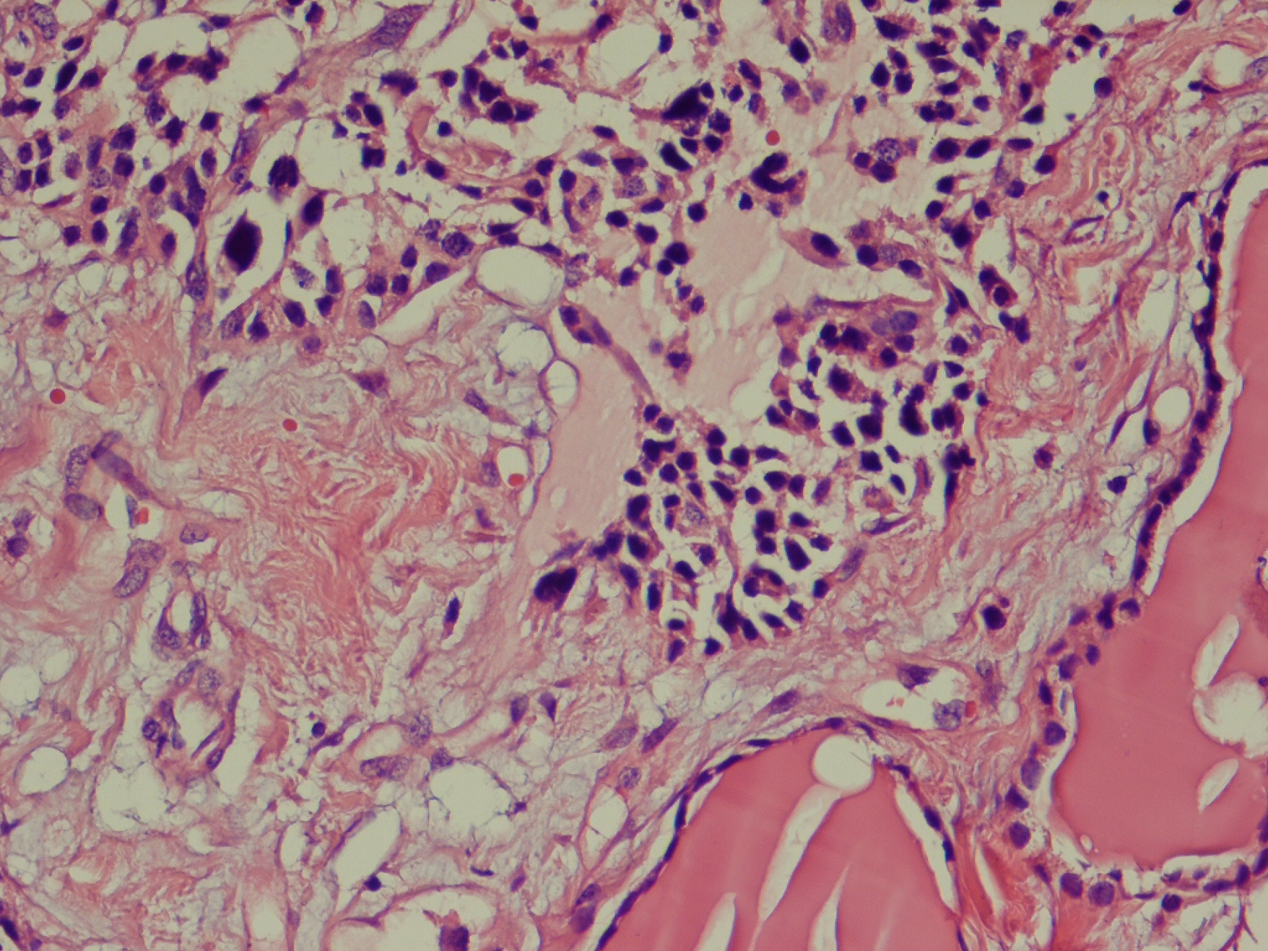
**Sheet of polygonal cells with round to elongated nuclei and clumped chromatin embedded in amorphous eosinophilic material, adjacent to normal thyroid follicles (hematoxylin and eosin [H&E] ×100)**.

**Figure 2:**
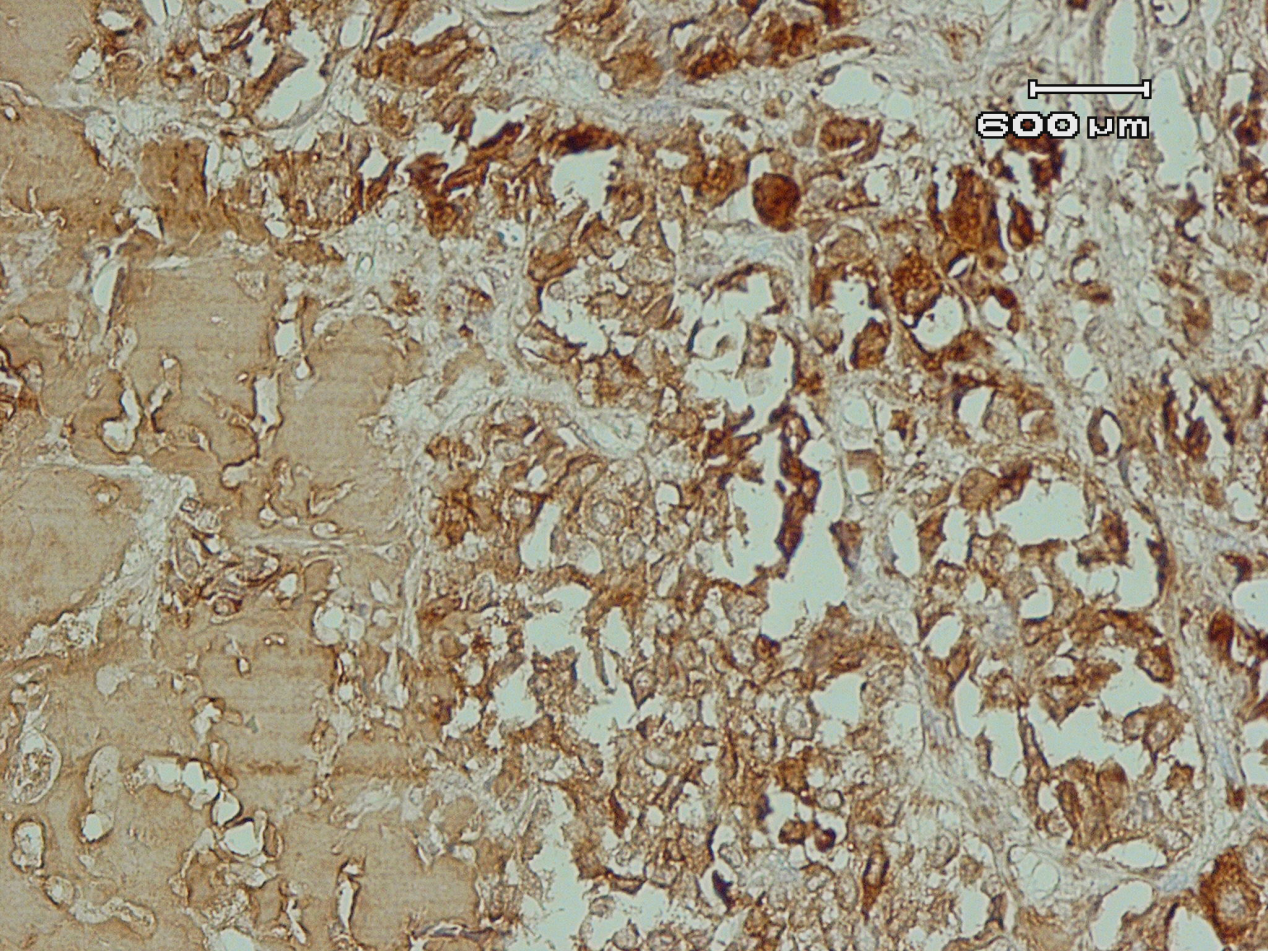
**Polygonal cells show positive cytoplasmic immunostaining for calcitonin (immunohistochemistry ×100)**.

In light microscopy, two lesions had strikingly different morphologies. The largest nodule consisted of a sheet-like growth of cells with round nuclei and clumped chromatin with scant amphophilic cytoplasm (Figure 1). Mitotic activity was low, and no area of necrosis or hemorrhage was observed. The stroma contained a homogeneous and pink ground substance. There was no evidence of C-cell hyperplasia in the rest of the normal thyroid. Tumor cells were immunoreactive for calcitonin (Figure 2), chromogranin A, and synaptophysin and were negative for thyroglobulin and cytokeratin.

**Figure 3:**
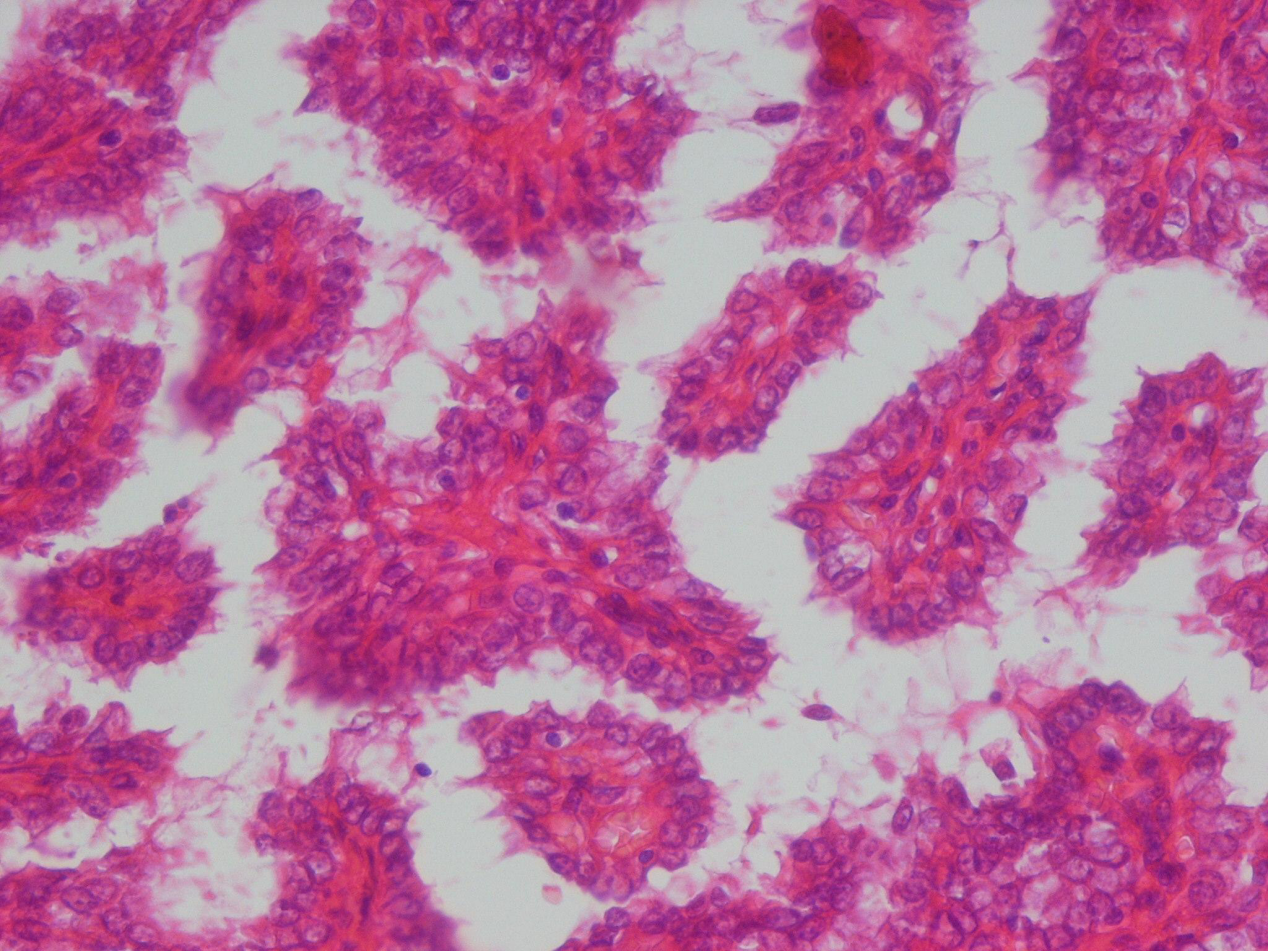
**Papillary structure lined by cuboidal to columnar cells with eosinophilic cytoplasm and occasional nuclear inclusions (hematoxylin and eosin [H&E] ×100)**.

The lesion located in the lower pole of the same lobe showed a papillary growth pattern with nuclear clearing, nuclear grooving, and occasional pseudoinclusions (Figure 3). These cells were immunoreactive for thyroglobulin and cytokeratin and were negative for calcitonin, chromogranin A, and synaptophysin. Four lymph nodes isolated from the neck dissection specimen showed metastasis of MTC, and one of them showed metastatic PTC. The stage of this tumor was T2N1M0. The result of a genetic analysis of RET oncogene was negative. For ablation of remnants of thyroid tissue, our patient received 150 mCi I^131^. Six months later, a whole-body scan with I^131 ^was performed in order to find metastatic or active thyroid tissue, but the result was negative. Suppression therapy with thyroid hormone was performed; during follow-up, the serum levels of thyroglobulin (normal range is not more than 60 μg/L), anti-Tg Ab (normal range is less than 2 IU/mL), and calcitonin (normal range is less than 11.5 ng/L) were routinely checked. The serum level was undetectable after surgery, and no increase in these parameters was detected. Our patient is clinically well 12 months after surgery.

## Discussion

The simultaneous occurrence of MTC and PTC in the same thyroid is a rare phenomenon that can be observed in two main settings: a mixed tumor showing dual differentiation [15] or a collision tumor (that is, a tumor with two separate and different components) [2-8]. Our case belongs to the latter category since lesions with features of MTC and PTC were detected in two different locations and separated by normal thyroid tissue.

Histopathology and immunohistochemical findings of the first nodule suggested that it was a small-cell variant of MTC. Histopathology and immunohistochemical findings of these nodules were small-cell variants of MTC and papillary microcarcinoma according to the current World Health Organization classification of thyroid tumors.

Our strategy in the treatment of thyroid carcinoma is similar to guidelines used in Western countries [16]. All patients were routinely examined by preoperative ultrasonography to estimate intrathyroid spread of the tumor. In patients with clinically involved lateral cervical lymph nodes detected by ultrasonography or computed tomography, a modified radical neck dissection was performed. For evaluation of bone and distant metastases, radioactive iodine whole-body scanning was done. Before radioactive iodine scanning, any thyroxin treatments were discontinued for four weeks and patients were placed under strict restriction of iodine-containing food for two weeks. Postoperative radioiodine therapy and thyroid-stimulating hormone suppressive therapy were performed. During follow-up, the serum levels of thyroglobulin, anti-Tg Ab, and calcitonin were routinely checked.

The coexistence of PTC and MTC has been reported in the literature [1-11]. These tumors occurred together more frequently in women, presented with a palpable neck mass, and were treated with surgery. Metastatic foci of either PTC or MTC were detected in few patients [4,6,7]. These lymph node metastases show pure tumor cell populations of one or two components or an admixture of both components within the same lymph node [9,17]. Distant metastases were described mostly in the mediastinum, lung, liver, and bone [18]. Fugazzola and colleagues [3] reported the familial clustering for these types of tumors, but the exact pathogenesis of these thyroid malignancies is completely unknown. Genetic analysis of RET oncogene in reported cases had conflicting results. Brauckhoff and colleagues [19] and Papi and colleagues [20] reported that germline point mutation of the RET gene had a potential role in the development of both MTC and PTC. However, according to Cerrato and colleagues [21], half of sporadic MTCs do not carry RET mutations and other genes, such as RB (retinoblastoma) and TP53 tumor suppressor pathways, may be involved in MTC formation. Rossi and colleagues [1] reported that both the RET and BRAF genes had a role in the genesis of the medullary-papillary collision tumors. The RET proto-oncogene plays a key role in the development of MTC. Vantyghem and colleagues [10] reported 11 cases of familial MTC-PTC according to clinical, histologic, or family features (or a combination of these features), but no RET defects were present. The authors suggested that another gene or uncommon abnormality of the RET gene was responsible for tumorgenesis. A recent animal study by Miller and colleagues [22,23] suggested that the PI3K or Ras signaling cascade alone was unable to transform thyroid follicular cells but that simultaneous activation had invasive and metastatic potential. Overall, the molecular evidence suggested that the two components of these heterogeneous groups of tumors were not derived from a common stem cell [23,24]. The origin of each carcinoma is embryologically different because the C cells stem from an ultimobranchial body that derived from the fourth pharyngeal pouch, whereas the thyroglobulin and thyroid hormone-producing cells come from the follicular epithelial cells derived from a median endodermal anlage from the tongue.

## Conclusions

In this case, the simultaneous occurrence of MTC and PTC presented as two distinct and well-defined tumor components. MTC shows a wide spectrum of morphological variants that resemble follicular, papillary, and undifferentiated carcinoma. The immunohistochemical studies for calcitonin along with thyroglobulin negativity usually confirm the C-cell origin of tumor cells. Overall, mixed MTC-PTC is a rare clinical entity and should be considered in the differential diagnosis of thyroid tumors, particularly in patients with a family history of thyroid malignancy.

## Abbreviations

Anti-Tg Ab: anti-thyroglobulin antibody; MTC: medullary thyroid carcinoma; PTC: papillary thyroid carcinoma.

## Consent

Written informed consent was obtained from the patient for publication of this case report and any accompanying images. A copy of the written consent is available for review by the Editor-in-Chief of this journal.

## Competing interests

The authors declare that they have no competing interests.

## Authors' contributions

NA participated in histology-related issues and literature review and drafted the manuscript. MSA contributed to patient treatment and revised respective sections in the manuscript. Both authors read and approved the final manuscript.
